# Leaf chlorophyll fluorescence and reflectance of oakleaf lettuce exposed to metal and metal(oid) oxide nanoparticles

**DOI:** 10.1186/s12870-023-04305-9

**Published:** 2023-06-21

**Authors:** Andrzej Kalisz, Andrzej Kornaś, Andrzej Skoczowski, Jakub Oliwa, Rita Jurkow, Joanna Gil, Agnieszka Sękara, Andrzej Sałata, Gianluca Caruso

**Affiliations:** 1 Department of Horticulture, University of Agriculture in Krakow, 29 Listopada 54, 31-425 Kraków, Poland; 2 Institute of Biology, Pedagogical University of Krakow, Podchorążych 2, 30‐084 Kraków, Poland; 3 Department of Vegetable and Medicinal Plants, University of Life Sciences in Lublin, Akademicka 15, 20-950 Lublin, Poland; 4 Department of Agricultural Sciences, University of Naples Federico II, 80055 Portici Naples, Italy

**Keywords:** *Lactuca sativa* L. var. *foliosa*, Non-organic nanoparticles, O-J-I-P, Photosystem II, Reflectance

## Abstract

**Background:**

Most nanoparticles (NPs) have a significant impact on the structure and function of the plant photosynthetic apparatus. However, their spectrum of action varies significantly, from beneficial stimulation to toxicity, depending on the type of NPs, the concentration used and plant genotypic diversity. Photosynthetic performance can be assessed through chlorophyll *a* fluorescence (ChlF) measurements. These data allow to indirectly obtain detailed information about primary light reactions, thylakoid electron transport reactions, dark enzymatic stroma reactions, slow regulatory processes, processes at the pigment level. It makes possible, together with leaf reflectance performance, to evaluate photosynthesis sensitivity to stress stimuli.

**Results:**

We investigated effects of different metal and metal(oid) oxide nanoparticles on photosynthesis of oakleaf lettuce seedlings by monitoring the chlorophyll *a* fluorescence light radiation and reflectance from the leaves. Observations of ChlF parameters and changes in leaf morphology were carried out for 9 days in two-day intervals. Spectrophotometric studies were performed at 9^th^ day. Suspensions of NPs with the following concentrations were used: 6% TiO_2_, SiO_2_; 3% CeO_2_, SnO_2_, Fe_2_O_3_; 0.004% (40 ppm) Ag; 0.002% (20 ppm) Au. Nanoparticles were applied directly on the leaves which caused small symptoms of chlorosis, necrosis and leaf veins deformation, but the plants fully recovered to the initial morphological state at 9^th^ day. Leaf reflectance analysis showed an increase in FRI for SiO_2_-NPs and CeO_2_-NPs treatments and ARI2 for Fe_2_O_3_, however, WBI and PRI coefficients for the latter nanoparticle were lower than in control. Chlorophyll *a* fluorescence parameters have changed due to NPs treatment. Fe_2_O_3_-NPs caused an increase in F_v_/F_0_, PI_ABS_, ET_0_/RC, DI_0_/RC, ABS/RC in different time points in comparison to control, also Ag, Au and SnO_2_ treatment caused an increase in F_v_/F_0_, PI_ABS_ or ET_0_/RC, respectively. On the other hand, TiO_2_-NPs caused a decrease in F_v_/F_m_ and F_v_/F_0_ parameters, but an increase in DI_0_/RC value was observed. SnO_2_-NPs decreased PI_ABS_, but increased ET_0_/RC than compared to control. Nanoparticles affected the shape of the O-J-I-P curve in slight manner, however, further analyses showed unfavourable changes within the PSII antenna, manifested by a slowdown in the transport of electrons between the Chl molecules of the light-harvesting complex II and the active center of PSII due to NPs application.

**Conclusion:**

Changes in ChlF parameters and leaf reflectance values clearly proved the significant influence of NPs on the functioning of the photosynthetic apparatus, especially right after NPs application. The nature of these changes was strictly depended on the type of nanoparticles and sometimes underwent very significant changes over time. The greatest changes in ChlF parameters were caused by Fe_2_O_3_ nanoparticles, followed by TiO_2_-NPs. After slight response of O-J-I-P curves to treatment of the plants with NPs the course of the light phase of photosynthesis stabilized and at 9^th^ day were comparable to the control curve.

## Background

Nanotechnology involves materials and objects from 1 to 100 nm in size [1], that have found wide application, among others in cosmetics, pharmaceuticals, personal-care products, paints, coatings, textiles, electronics, environmental remediation, food production and food packaging [2, 3]. Nanotechnology offers humans plenty of benefits along with the new challenges towards the safety of the environment and human health. The production, wide use, accidental or intentional disposal of nanomaterials will inevitably lead to their release into the atmosphere, water, and soil [4, 5]. The challenges posed by nanomaterials are to determine how their physical and chemical properties differ from conventional materials and whether they may have potential harmful effects on the environment and on biota [6, 7]. Interactions between plants and engineered nanoparticles (NPs) may lead to influence the plant physiology and possibly food chain security, and represent one of the most important problems that must be faced concerning rapid development of nanotechnology [8]. Plants exposed to NPs may show positive or negative responses in growth, physiological processes (like photosynthesis) and biochemical pathways [9–12]. Usually, at higher concentrations, NPs negatively affect plants causing abiotic stress consisting of significant impairment of photosynthesis, generating reactive oxygen species, damaging cellular membranes, proteins and nucleic acids and inducing genotoxicity; however, some NPs could be used to alleviate the effects of different stresses on plants in a dose-dependent manner [13, 14]. Also, plant species are key players of the net outcome arising from NPs–plant interactions [15]. Ghorbanpour et al. [12] pointed to possibility of promoting photosynthesis in plants treated with a chosen dose of a given type of NPs via enhancing chlorophyll content, increasing the activity of RuBisCO enzyme, improving the performance of photosystem II, and CO_2_ assimilation, as well as broadening the chloroplast photoabsorption spectrum.

The influence of nanoparticles on the photosynthesis is a subject of ongoing research. Application of silica nanoparcticles (Si-NPs) was reported to enhance photosynthesis in wheat and lupin together with an increase in the amount of chlorophyll [16]. Titanium dioxide nanoparticles (TiO_2_-NPs) applied onto *Arabidopsis thaliana* seedlings caused photosynthesis improvement, which was probably connected with significant increase of light-harvesting complex II (LHCII) activity and LHCII content on the thylakoid membrane [17]. An increase in net photosynthetic rate due to TiO_2_-NPs application on *Mentha piperita* L. was confirmed by Ahmad et al. [18]. In spinach plants, TiO_2_-NPs promoted the light-dependent phase of photosynthesis [19], whereas in tomato, this process was negatively affected [20]. Lu et al. [21] reported generation of excessive hydroxyl radical (^•^OH), facilitated the degradation of chlorophyll and posting a negative impact on the photosynthesis in wheat plants treated with Fe_2_O_3_-NPs. Da Costa and Sharma [22] described that photosynthetic rate and photosynthetic pigment contents declined in rice treated with CuO-NPs. According to Kataria et al. [23], NPs either boost up the photosynthesis processes by improving light-harvesting complexes in plants or hinder their pathways by blocking electron transport chain and they affect photosynthetic rate by change in expression several genes and activity enzymes like carbonic anhydrase, ribulose bisphosphate carboxylase-oxygenase (RuBisCO) and phospho*enol*pyruvate (PEP) carboxylase. More information about influence of NPs on photosynthetic apparatus and photosynthetic process can be found in reviews of Tighe-Neira et al. [15] and Ghorbanpour et al. [12].

Many of photosynthetic components and reactions (photosynthetic pigments for light absorption, photosystems and the light reactions for NADPH and ATP generation, and the dark reactions [Calvin–Benson–Bassham or C3 cycle] for CO_2_ assimilation) are significantly affected by different abiotic stresses which in consequence reduce the growth, development and yield of plants [24]. Measured signals of chlorophyll *a* fluorescence (ChlF) are used to determine photosynthetic efficiency [25] that allows to estimate the energy absorption by the pigments of the antenna system, the capture of an excitation by the reaction centre, and the subsequent electron transport to the final electron acceptor [26]. Measurements the fluorescence transient (O-J-I-P) by JIP test make possible to quantify the flux of energy passing through the photosystems, to evaluate the photosynthetic performance of plants, and to analyse the PSII operation [27, 28]. ChlF technique is also non-destructive and precise tool to predict, monitor, and identify stress in plants caused by different environmental factors, e.g. heat and low temperature, high or low light intensity, drought, salinity, nutrient deficiency, heavy metals and, potentially, by nanoparticles [25, 29, 30]. ChlF signals can be determined by single point measurements [31] and this technique is often used by researchers to determine alterations in photosynthetic activity of plants treated with NPs (e.g. [14, 32]. Another non-destructive method in plant research is the measurement of leaf light reflectance, the unique leaf reflectance signatures serve as indicators of environmental stress [33, 34]. Moreover, reflectance analysis allows to determine changes in chemical composition of the leaves (including pigment system restructuring) and the degree of light energy utilization [35].

In our earlier experiments we examined the effects of NPs on oakleaf lettuce, with particular emphasis on plant antioxidative mechanisms and biochemical response [36, 37]. In this report, we investigated effects of different metal nanoparticles and metal(oid) oxide nanoparticles on photosynthesis of oakleaf lettuce seedlings by the use of chlorophyll fluorescence and reflectance from the leaves data. We may assume that different nanoparticles may act in different manner on photosynthesis of oakleaf lettuce which can be proved via chlorophyll fluorescence measurements performed in our study; moreover, this may change over time.

## Results

### Morphological effects after treating plants with nanoparticles

**Fig. 1:**
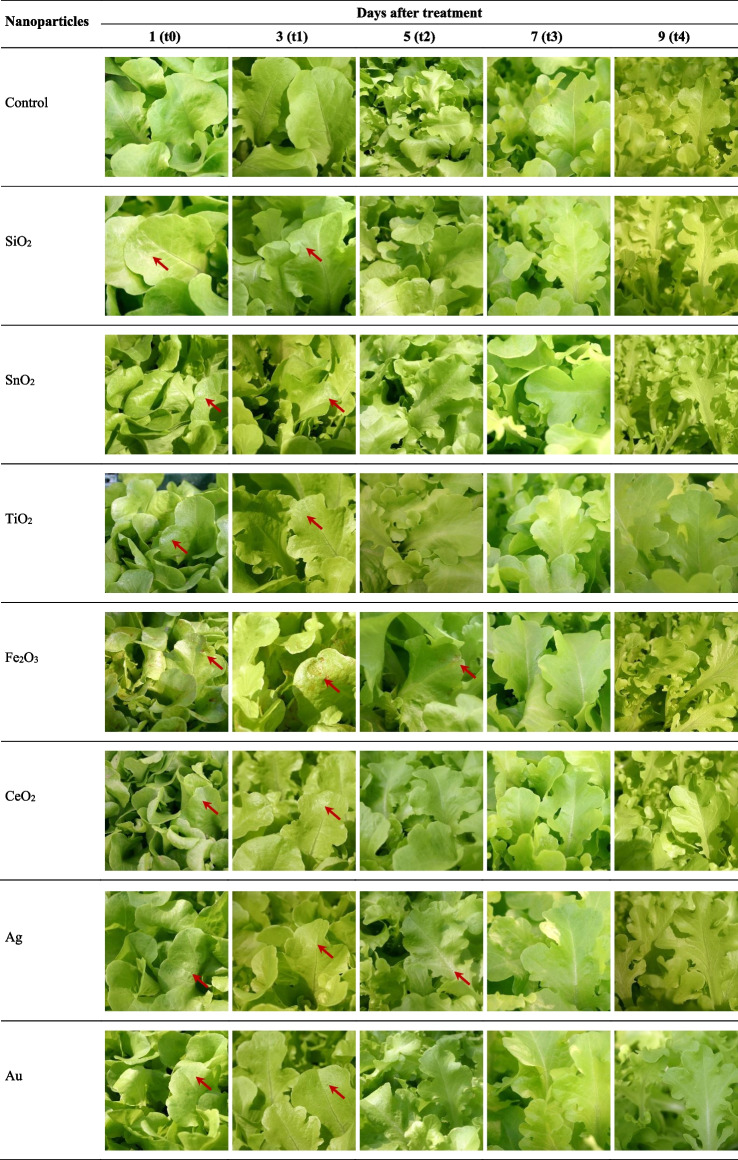
Leaves of oakleaf lettuce seedlings after treatment with different suspensions of non-organic nanoparticles. Arrows indicate morphological effects after treating plants with nanoparticles

Photographic documentation of the oakleaf lettuce seedlings was made one day after NPs application (t0), and next 3, 5, 7, 9 days (t1, t2, t3, t4, respectively) after NPs treatment (Fig. 1). Our intention was to monitor potential changes on the surface of seedling leaves, we were mainly interested in finding regular or irregular discolorations, necrosis and leaf deformations. Light discoloration appeared in some places on the lettuce leaves one day after the plants were treated with SiO_2_-NPs, small traces of damages were still visible on the leaves after two consecutive days, but they were less noticeable. A similar situation occurred when the plants were treated with SnO_2_-NPs and CeO_2_-NPs. Discolorations appeared on the leaves immediately after one day after they were sprayed with TiO_2_-NPs, some changes persisted up to 3^rd^ day, the leaves also had a slight metallic sheen. The strongest changes on the surface of lettuce leaves were noticed after the application of Fe_2_O_3_ nanoparticles. Chlorosis and necrosis appeared after one day, and persisted up to the 5^th^ day (t2) after spraying the plants with the suspension of that nanoparticles. The areas of damage to the leaf tissue had a distinct rusty colour. Very fine point changes were noted on the leaves treated by Au-NPs, which lasted for only a few days, in the case of Ag-NPs, slight changes on leaf surface and deformations of the main leaf nerve were observed, especially it was visible on the 5^th^ day (t2) after the application of that nanoparticles. It should be emphasized that on the 7^th^ or 9^th^ day (t3 and t4, respectively), in all treatments, damages have gradually decreased and disappeared, the leaf tissues fully recovered.

### Optical properties of leaves

**Fig. 2:**
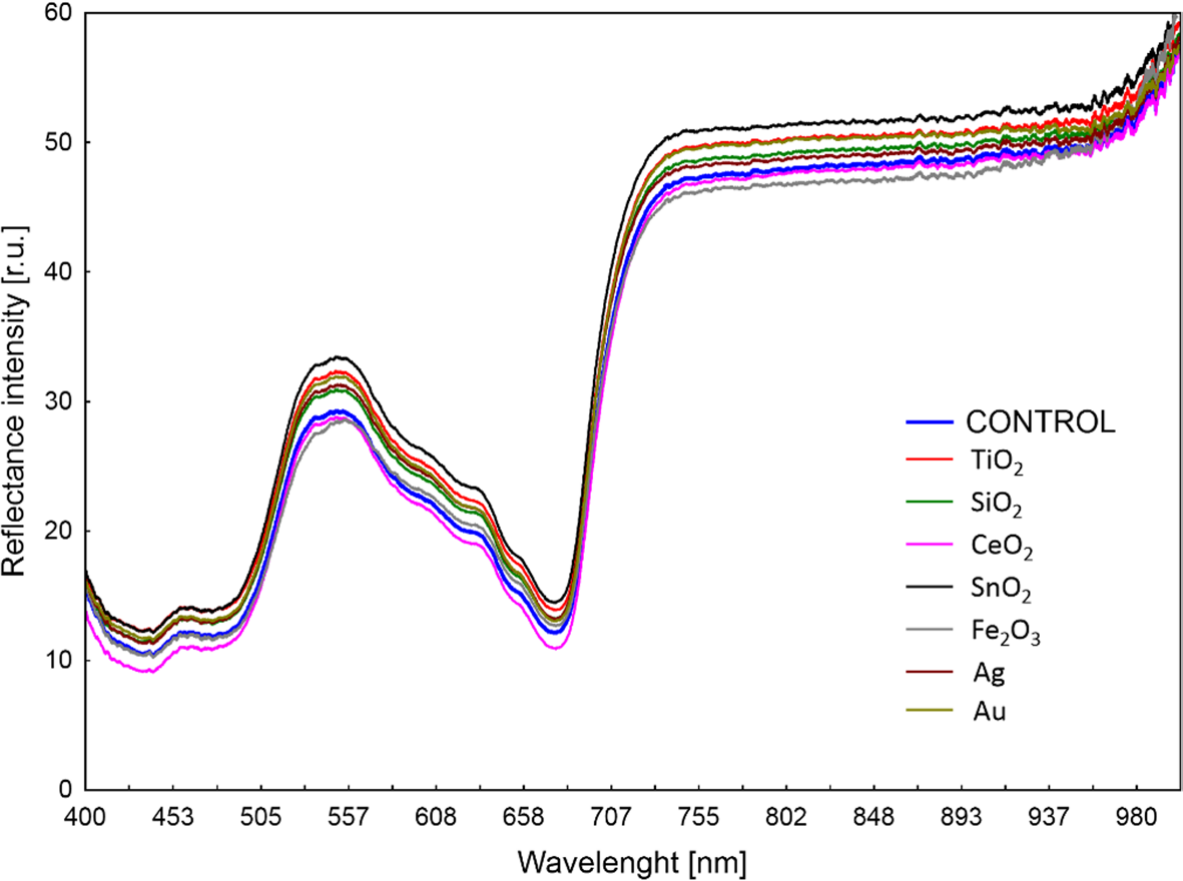
Light radiation reflectance curves of oakleaf lettuce leaves treated with suspensions of various nanoparticles. Each curve represents the average of 10 measurements. The results correspond to the 9^th^ day after treatment

The spectrum of reflection of lettuce leaves of all analysed treatments was characterized by a similar shape of the curves (Fig. 2). In terms of photosynthetic active radiation (PAR), the differences between the intensity of reflection in plants after individual nanoparticles treatment were rather small.

Anthocyanin Reflectance Index 2 (ARI2), was the highest in the leaves of oakleaf lettuce treated with Fe_2_O_3_-NPs (difference reached 95.2% as compared to control, on average) (Table 1). In the case of the remaining tested nanoparticles, the ARI2 was comparable to the control. The highest Flavonol Reflectance Index (FRI), in comparison to control plants, was demonstrated for leaves treated with SiO_2_-NPs or CeO_2_-NPs (differences reached 0.174 and 0.215 units, respectively). The FRI of other treatments was similar to that observed in control. The obtained Water Band Index (WBI) values for oakleaf lettuce leaves indicate relatively good hydration of tissues of studied treatments (Table 1). However, treatment with Fe_2_O_3_-NPs lowered by 3.8% and with Au increased by 1.9% leaf hydration relative to control. Higher Photochemical Reflectance Index (PRI) values indicate better efficiency of PAR utilization, however, there was no increase in the value of this parameter in any treatments compared to the control, outright contrary, the weakest use of PAR by oakleaf lettuce leaves was recorded after treatment with Fe_2_O_3_-NPs (decrease by 99.85% as compared to control). Structure Independent Pigment Index (SIPI), reflecting the ratio of carotenoids to chlorophyll *a* content, did not differ between treatments.

**Table 1:**
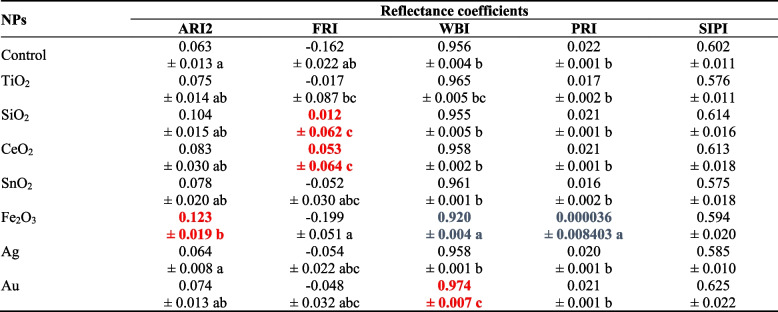
The values of the fluorescence coefficients in the leaves of oakleaf lettuce treated with solutions of various nanoparticles

Means in a column followed by different letters are significantly different at *p* ≤ 0.05 according to Duncan’s test. No letters denotes no significant differences. Bolded data in red show significantly higher level than control, while bolded data in blue represent lower values as compared to control treatment. Data are presented as means ± SD (*n* = 10)

### Chlorophyll *a* fluorescence measurements

In the present experiment, the analysis of ChlF parameters showed that applied types of nanoparticles affected significantly monitored parameters of ChlF and photosynthetic efficiency of oakleaf lettuce in time dependent manner (Table 2). At the t1 time point (3 days after NPs treatment), higher F_v_/F_0_ and PI_ABS_ values than in the control plants were observed for the plants subjected to Fe_2_O_3_-NPs (in the case of both parameters, it was an increase by 37.7% and 186.8%, respectively). Seedlings treated with Ag-NPs showed an increase by 27.0% in F_v_/F_0_ and Au-NPs caused an increase by 200.0% in PI_ABS_ parameter when compared to non-treated control plants. Rate of electron transfer by the active PSII reaction center (ET_0_/RC) increased due to SnO_2_-NPs treatment by 27.8%. However, after next few days all above mentioned changes returned to a level comparable to the control. At that t2 time point, there were significant increases in ET_0_/RC, DI_0_/RC and ABS/RC values in plants treated with Fe_2_O_3_-NPs (by 25.1%, 189.0% and 59.7%, respectively), while for seedlings subjected to SnO_2_-NPs value of PI_ABS_ significantly decreased (by 43.6%, on average) as compared to control. No significant differences in tested ChlF parameters were observed 7 days after treatments (t3) between control and NPs-treated plants, however, some differences occurred between particular NPs treatments. On the 9^th^ day (t4), plants treated with TiO_2_-NPs showed higher DI_0_/RC value, by 24.4%, on average, compared to control seedlings, which was accompanied by a simultaneous reduction in the values of F_v_/F_m_ and F_v_/F_0_ (by 3.5% and 13.5%, respectively). Absorption flux (of antenna Chls) per RC (ABS/RC) decreased by 9.9% at that time due to Ag-NPs treatment of the plants in comparison to control. There were no significant changes in 1- V_I_ parameter between control plants and plants treated with NPs. Some statistically significant differentiation occurred between particular NPs treatment, *inter alia* between Au-NPs and Fe_2_O_3_-NPs there was an increase in 1–V_I_ value for the latter by 26.4% (t2) and 15.2% (t4).

**Table 2:**
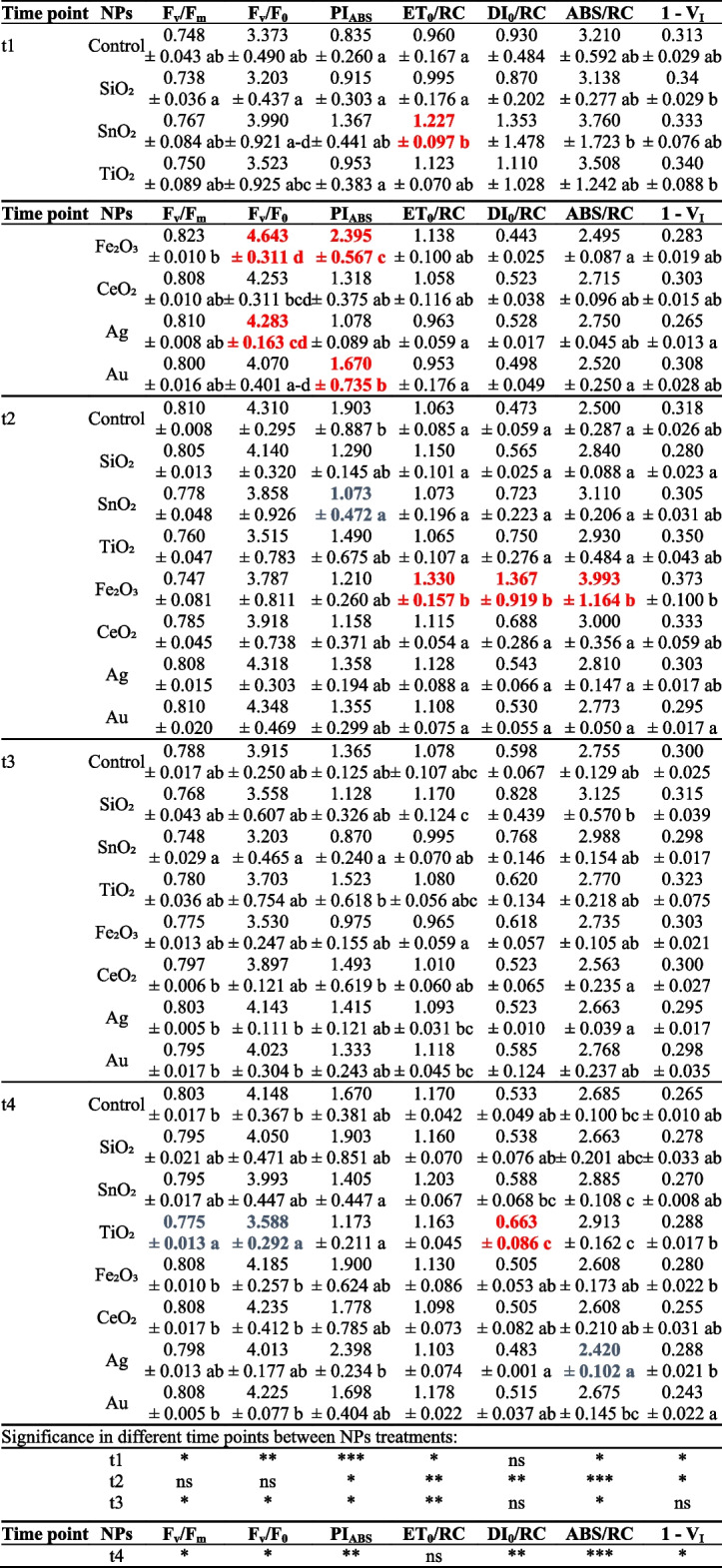
Selected parameters of chlorophyll *a* fluorescence of the leaves of oakleaf lettuce seedlings at 3, 5, 7, and 9 day after NPs treatment (t1, t2, t3, t4, respectively)

Means in a column, within each time point, followed by different letters are significantly different at *p* ≤ 0.05 according to Duncan’s test. No letters denotes no significant differences. Bolded data in red show significantly higher level than control, while bolded data in blue represent lower values as compared to control treatment. Level of significance: * *p* ≤ 0.05; ** *p* ≤ 0.01; *** *p* ≤ 0.001, ns – not significant. Data are presented as means ± SD (*n* = 4)

**Fig. 3:**
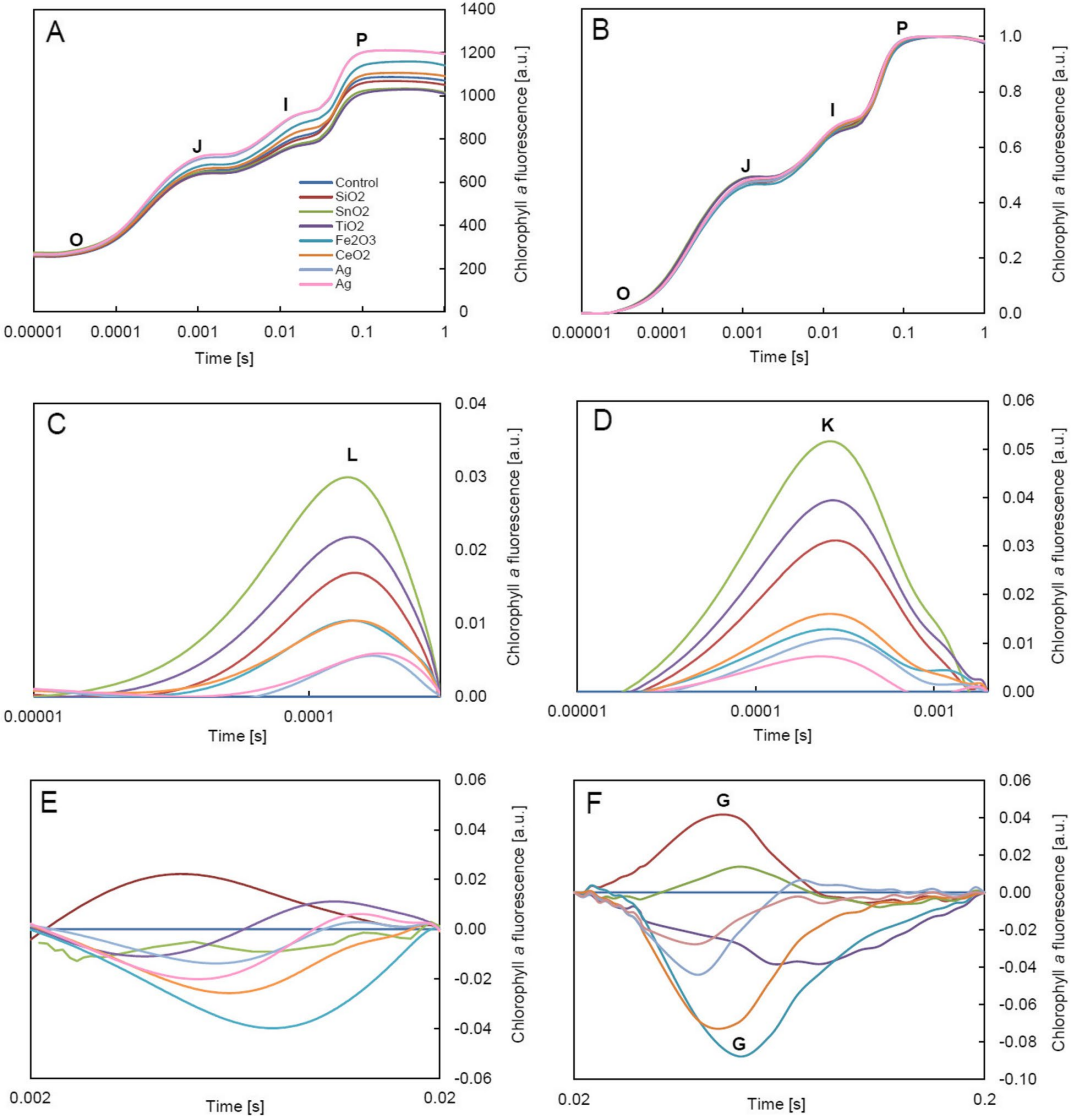
Chlorophyll *a* fluorescence induction curves (OJIP) of oakleaf lettuce leaves treated with nanoparticle suspensions and for control plants: **A** non-normalized curves, **B** curves after normalization to points O and P. Difference curves for individual sections: **C** O-K, **D** O-J, **E** J-I, **F** I-P, normalized to values corresponding to the characteristic points of the fluorescence induction curve. All curves were made on the basis of the mean values of the four measurement dates (t1, t2, t3, t4, i.e. 3, 5, 7, 9 days after NPs treatment, respectively)

**Fig. 4:**
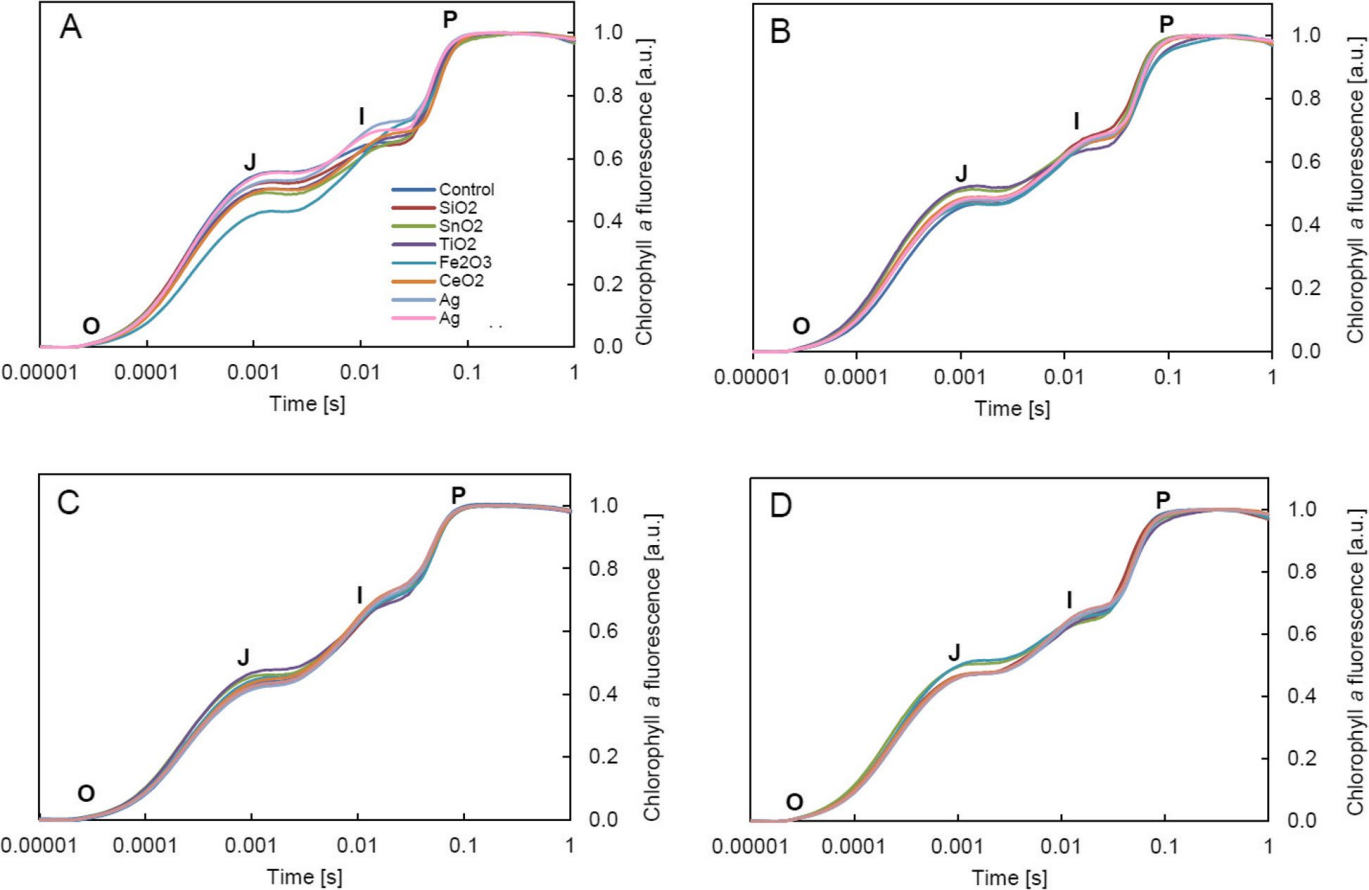
Chlorophyll *a* fluorescence induction curves (OJIP) of oakleaf lettuce leaves treated with nanoparticle suspensions and for control plants after normalization to points O and P for individual measurement dates: **A** t1, **B** t2, **C** t3, **D** t4 (i.e. 3, 5, 7, 9 days after NPs treatment, respectively)

The averaged Chl*a* fluorescence induction curves (t1-t4) showed the presence of typical points O-J-I-P and a rapid increase in the ChlF intensity in the I-P phase (Fig. 3A-B). The minimum fluorescence was similar in all plants, however, treatment with NPs resulted in fluctuations in the maximum fluorescence (Fig. 3A). The greatest increase in F_m_ was observed after treatment with Ag-NPs. The analysis of normalized O-J-I-P curves showed no significant disturbances in electron transport in PSII in NPs-treated plants (no significant changes in the shape of the curves – Fig. 3B). However, the calculated differential curves (Fig. 3C-F) revealed the presence of stress-bands, thanks to which it was possible to evaluate even subtle changes in the efficiency of electron chain. The L- and K-bands (Fig. 3C-D) indicated that treatment with NPs caused unfavorable changes mainly in the PSII antenna. The highest intensity of ChlF in L-band and K-band was observed in plants with SnO_2_-NPs. High fluorescence intensity in these bands was also noted after the application of TiO_2_-NPs and SiO_2_-NPs. In the remaining treatments, disturbances in the initial stages of energy transport through the PSII were insignificant. In turn, the negative G-band (Fig. 3F) revealed disturbances in the reduction of electron carriers on the acceptor side of PSI, especially in leaves treated with Fe_2_O_3_-NPs and CeO_2_-NPs. All changes observed were temporary (Fig. 4A-D). The most visible response of the photosynthetic apparatus to NPs occurred at time t1, when in the O-J phase the greatest deviations in the ChlF intensity (compared to the control) were observed (Fig. 4A). The analysis of changes over time (t1-t4) showed that after the initial, slight response of the leaves to the treatment with nanoparticles, the course of the photosynthesis light phase was stabilized and at time point t4, there were no significant differences between the NPs-treated plants and the control plants (Fig. 4D).

**Fig. 5:**
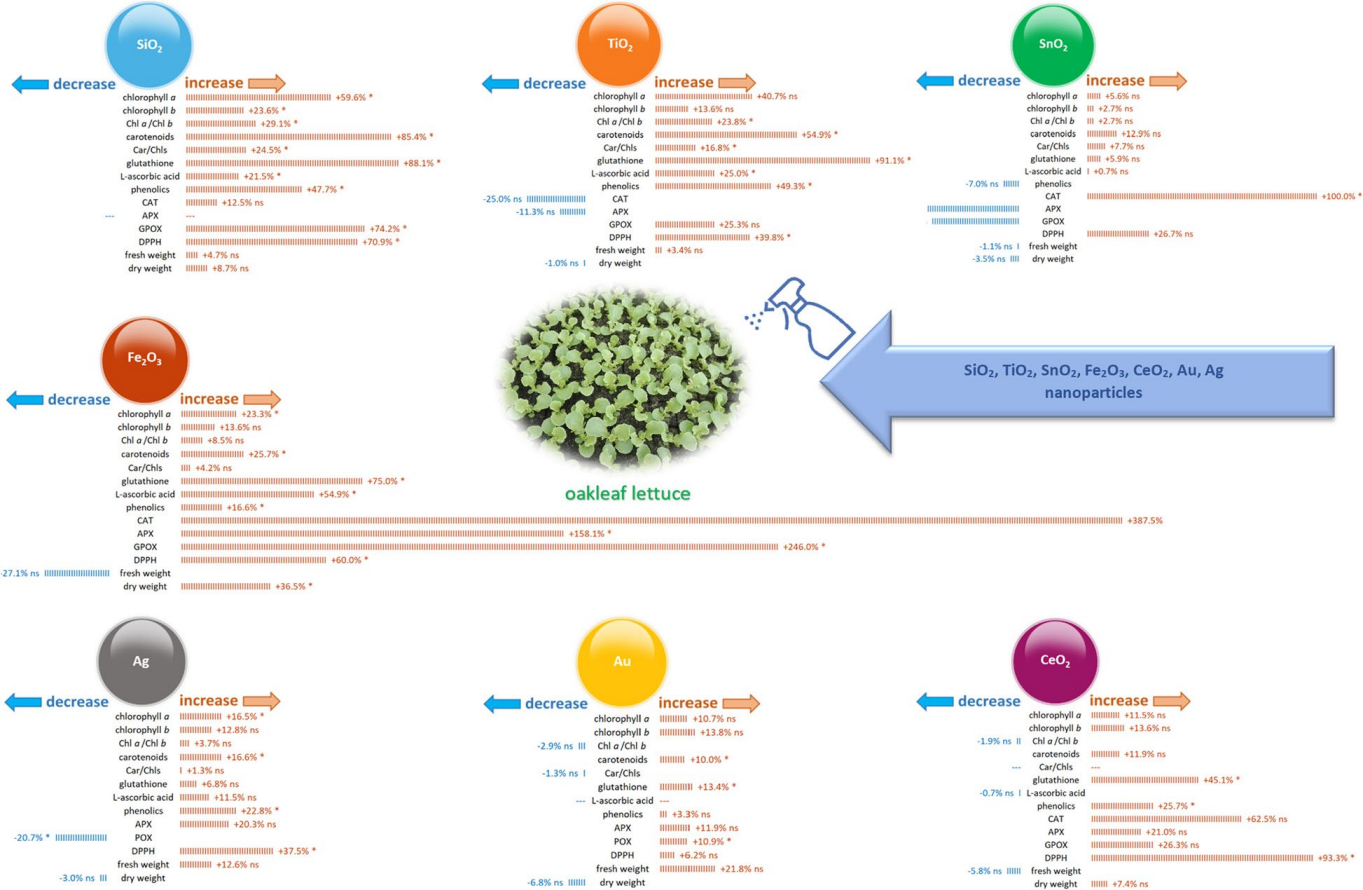
Changes in chlorophyll pigments, antioxidants, fresh weight and dry weight of oakleaf lettuce plants treated with SiO_2_, TiO_2_, SnO_2_, Fe_2_O_3_, CeO_2_, Au and Ag nanoparticles. Data adapted from Jurkow et al. [36] and Jurkow et al. [37] for the same concentrations of NPs as used in the present study. Plant samples for laboratory analyses were taken 7 days after NPs treatment. Significant changes were marked by asterisks

Our previous research focused on biochemical changes in oakleaf lettuce seedlings treated with nanoparticles [36, 37]. The present study is a complementary continuation of these reports, thus we decided to include here an infographic (Fig. 5), showing gathered together data from published reports. The same concentrations of the same NPs were selected as in the present study. Data, presented as percentage increase or decrease, visualize changes caused by NPs in the activity of antioxidant enzymes and in the content of several bioactive compounds, with particular emphasis on chlorophylls and carotenoids. We believe that such a holistic approach will facilitate better understanding alterations in parameters observed in the present study. As can be seen on Fig. 5, foliar application of all nanoparticles caused an increase in chlorophyll *a*, chlorophyll *b* and carotenoids contents. Chlorophyll *a* to chlorophyll *b* ratio (Chl *a*/Chl *b*) increased in most cases with an exception for CeO_2_-NPs and Au-NPs. Carotenoids to chlorophylls ratio (Car/Chls) decreased only for Au-NPs or stayed unchanged for CeO_2_-NPs and increased in other treatments when compared to control. Activity of several antioxidant enzymes – ascorbate peroxidase (APX), guaiacol peroxidase (GPOX), catalase (CAT), total peroxidases (POX) – increased due to application of SiO_2_-NPs (CAT, GPOX), TiO_2_-NPs (GPOX), SnO_2_-NPs (CAT), Fe_2_O_3_-NPs and CeO_2_-NPs (CAT, APX, GPOX), Au-NPs (APX, POX) and Ag-NPs (APX). In some other cases, activity of the enzymes was similar or became even lower to that of control plants. Content of glutathione (GSH), L-ascorbic acid, total phenolics increased due to SiO_2_-NPs, TiO_2_-NPs, Fe_2_O_3_-NPs, and Ag-NPs application, total phenolics level decreased in the plants of SnO_2_-NPs treatment, while L-ascorbic acid level stayed unchanged for the plants treated with CeO_2_-NPs and Ag-NPs. Some nanoparticles positively influenced plant growth, which could be seen through the increase in fresh weight (FW), i.e. metal NPs like Au and Ag increased fresh weight by 21.8% and 12.6%, respectively, while others not – there was even a decrease in FW that reached 27.1% after Fe_2_O_3_ application in comparison to control. On the other hand, the highest increase in dry weight DW was noted for Fe_2_O_3_-NPs (36.5%), while the greatest decline showed plants treated with Au-NPs (6.8%). The most prominent alterations in investigated parameters occurred for Fe_2_O_3_-NPs, it was spectacular especially for activities of antioxidant enzymes (increases up to 387.5%).

## Discussion

Monitoring of morphological changes on the surface of the plants’ leaves treated with the tested nanoparticles confirmed their phytotoxicity effects on plants in this aspect. The symptoms of leaf damages appeared already one day after NPs application and were visible until the 3^rd^ day in all experimental treatments. In the case of Ag-NPs and Fe_2_O_3_-NPs they were observed even longer, up to 5^th^ day. However, the damages were of limited extent and the plants were capable to regenerate damaged tissues and fully recovered to initial state. Finally, a week after the application of nanoparticles, all of the plants did not show on their leaves any external signs of NPs phytotoxicity. The observed changes on the leaves were for us the first signal that NPs may act effectively on the plants more broadly, not only on leaf morphology, which was confirmed by various other measurements carried out during the experiment.

Analysis of reflectance indices of light radiation from leaves depending on the used nanoparticles is a valuable source of information about changes in the content of plant pigments [38]. Higher ARI2 in oakleaf lettuce treated with Fe_2_O_3_-NPs is not consistent with the results of Kiapour et al. [39] who noted that level of anthocyanins in roselle plants treated with Fe_2_O_3_-NPs did not significantly change in relation to control. On the other hand, our previously published data ([37], Fig. 5) pointed to an increase in phenolic compounds in lettuce plants treated with Fe_2_O_3_-NPs, which indicates important role of phenolic compounds in plant antioxidant systems. An increase in FRI value in oakleaf lettuce treated with CeO_2_-NPs is similar with results of our earlier research on butterhead lettuce and sweet pepper treated with the same nanoparticles [40], however, content of flavonoids in plants treated with SiO_2_-NPs was lower (butterhead lettuce) or similar (sweet pepper) to the control. The WBI values in plants usually range from 0.8 to 1.2 [41], thus it was typical for oakleaf lettuce in our study. The PRI coefficient is correlated with zeaxanthin (de-epoxidation in the xanthophyll cycle) and the effectiveness of PAR utilization by plants [42]. Gamon et al. [43] showed that by using PRI index it is possible to track changes in effectiveness of light radiation use in the photosynthesis process by plants affected by various environmental factors (e.g., availability of mineral substances). In our study, the weakest use of PAR by oakleaf lettuce leaves was recorded for the plants subjected to Fe_2_O_3_-NPs where a decrease by almost 100% in the value of PRI index was observed in comparison to control. Although fluorescence coefficient SIPI (carotenoids to chlorophyll *a* ratio) was not affected by nanoparticles in the present study, our previous data showed that carotenoids to total chlorophylls ratio can be changed due to NPs treatment (Fig. 5). The applications of all NPs had a significant effect on the contents of carotenoids (Fig. 5). The best results obtained with applications of SiO_2_-NPs, TiO_2_-NPs and Fe_2_O_3_-NPs, there was an increase in carotenoids content by 85.4%; 54.9% and 25.7%, respectively, when compared to control plants. Carotenoids have various functions in plants, they function as accessory pigments for light harvesting and as photoresists during photosynthesis, in addition to being antioxidants [44]. Therefore, the increase in these pigments in plants is a favourable response, since it can be directly related to the increase in antioxidant capacity [45]. It is possible that observed increase of carotenoids in NPs-treated plants was due to the activation of the antioxidant defense system of the oakleaf lettuce.

Chlorophyll *a* fluorescence (ChlF) can be used as a probe of photosynthetic efficiency, reflecting the impacts of environmental factors and changes in the physiological state of the plants [46]. An influence of nanoparticles on photosynthetic apparatus has been also described by Tighe-Neira et al. [15] in their review. Several scientific reports have shown effects of NPs on photosynthesis of the plants, observed by alterations in the ChlF parameters, these effects can be both negative or positive. For example, Elshoky et al. [14] subjected pea (*Pisum sativum*) plants to ZnO-Si-NPs and ZnO-NPs and observed that 200 mg L^−1^ ZnO-NPs did not influence the functions of both photosystems, while 400 mg L^−1^ ZnO-Si-NPs had beneficial effects on the effective quantum yield of photosystem II (PSII) and the photochemistry of photosystem I (PSI). This confirms the usefulness of the chlorophyll *a* fluorescence analysis in the evaluation of photosynthetic efficiency.

In our study, Fe_2_O_3_-NPs had the strongest effects on plants, which was particularly visible in the alterations of ChlF parameters 3 days and 5 days after NPs treatment. Fe_2_O_3_-NPs caused the increase in F_V_/F_0_, which is the ratio between the rate constants of photochemical and nonphotochemical deactivation of excited Chl molecules [47–49]. This parameter can be taken as stress indicator, but it can suggest, together with other ChlF parameters, that the plants treated with Fe_2_O_3_-NPs could regain a higher activity of reaction centers. Tombuloglu et al. [50] investigated the impact of hematite nanoparticles (α-Fe_2_O_3_-NPs) on barley and showed a significant decline in the maximum quantum efficiency of PSII photochemistry (F_v_/F_m_) in treated plants, however, the treatment led to an increase in measurement ratio of plant efficiency which represents the amount of energy used in photochemistry by PSII and to an increase in photosynthetic electron transport rate, which is also valuable stress indicator, compared to the control. On the other hand, Kreslavski et al. [51] noted, that the values of maximum quantum yield (F_v_/F_m_) were higher in wheat treated with Fe_3_O_4_-NPs and Fe_2_O_3_-NPs than in the control. Any significant changes in F_v_/F_m_ parameter in the plants subjected to Fe_2_O_3_-NPs were not observed in our experiment with oakleaf lettuce, however, a decrease in F_v_/F_m_ was noted for the plants treated with TiO_2_-NPs. A decrease in F_v_/F_m_ value indicated that the light absorbed by the plants that was used in photosynthesis was reduced. The change in the active PSII reaction centers (F_v_/F_0_) was similar to that of F_v_/F_m_ for plants treated with TiO_2_-NPs, it was lower than in control plants, however, higher energy dissipation (DI_0_/RC) was observed. As revealed from the study of Ahmad et al. [18], F_v_/F_m_ parameter exhibited a significant enrichment of 7.2% when mint (*Mentha piperita*) plants were treated with 100 mg L^−1^ TiO_2_-NPs as compared to control. It means that TiO_2_-NPs can have positive effects on photosynthetic efficiency as they can increase the energy and number of electrons in the transport chain [52], and also water photolysis and ATP formation [53]. In our case, however, TiO_2_-NPs revealed a negative effect on photosynthetic efficiency. Our data showed also that Fe_2_O_3_-NPs treatment increased the performance index (PI_ABS_). The performance index can serve as an index of plant/variety vitality and/or sensitivity to abiotic stress [54], moreover, PI_ABS_ reflects the functionality of both photosystems I and II and gives quantitative information on the current state of plant performance under stress conditions [27]. Higher PI_ABS_ for plants treated with Fe_2_O_3_-NPs, noted in present study, indicated that the potential PSII activity, photosynthesis photoinhibition, and PSII function were not damaged. The increasing PI_ABS_ value by Fe_2_O_3_-NPs treatment may be also related to the increase in the density of the active reaction centers of PSII [55]. Higher PI_ABS_ observed for Fe_2_O_3_-NPs plants in comparison to control should be discussed with higher ET_0_/RC, DI_0_/RC and ABS/RC values, also noted for that plants. These parameters pointed to electron transport flux per reaction center, energy dissipation and average photon absorption (effective antenna size of an active reaction center), respectively. Higher ET_0_/RC in plants treated with Fe_2_O_3_-NPs than in control pointed to lack of disturbance in light reactions in photosynthesis, an increase in DI_0_/RC indicated that most energies in RC dissipated in a form of heat due to self-protection when a plant was in stress conditions, while the ratio of ABS/RC increased due to inactivation of some active RCs. Our previous report also confirmed strong response of oakleaf lettuce to Fe_2_O_3_-NPs treatment that can be seen on Fig. 5 [37]. A certain surprise was the increase in the PI_ABS_ value for the plants treated with Au-NPs, with no significant changes in other measured parameters of chlorophyll *a* fluorescence. In this case, it should be regarded as a positive effect of Au-NPs on photosynthesis. According to Avellan et al. [56] Au-NPs increased the stomatal conductance and the net photosynthesis rate of the exposed wheat leaves. In our study, Ag-NPs treatment increased F_v_/F_0_ and decreased ABS/RC values in comparison to control. It means that photon absorption was reduced together with decrease in active RCs. According to Dewez et al. [57], Ag-NPs provided in duckweed (*Lemna gibba*) strong inhibitory effect on energy transfer from light harvesting complex to photosynthetic reaction centers, causing deterioration of the PSII water splitting system and inactivation of PSII reaction centers. Matorin et al. [58] examined the influence of Ag-NPs on the photosynthetic activity of *Chlamydomonas reinhardtii*. They found that Ag-NPs had no direct effects on PSI, but inhibited the electron transfer in PSII, and enhanced the production of secondary quinone electron acceptors (QB). An interesting case in our experiment were the plants treated with SnO_2_-NPs showing a decrease in PI_ABS_, which is a negative signal regarding photosynthesis. However, the ET_0_/RC parameter, which is usually reduced in stressed plants [59], it increased for oakleaf lettuce seedlings treated with SnO_2_-NPs, suggesting that the reaction of transfer of light energy to photosystem I was proceeding relatively efficiently by that plants.

The ChlF induction curve with fast chlorophyll fluorescence induction curve (O-J-I-P) steps obtained from different plants can show a significant change under nanoparticles treatment [32, 60]. The mechanisms underlying the regulation of photosynthesis by nanoparticles are related to decreasing or enhancing the chlorophyll content and electron transport rate, influencing the performance of PSII, and CO_2_ assimilation, causing damages to chloroplast components, broadening the chloroplast photoabsorption spectrum, regulating Hill reaction and Calvin cycle, changing the activity of key photosynthetic enzymes like Rubisco, moreover, light harvesting NPs (e.g. TiO_2_-NPs) may capture and transfer more electrons altering photosynthetic efficiency [12]. In our study, the analysis of fluorescence signals provides detailed information on the status and function of photosystem II (PSII) reaction centers, light harvesting antenna complexes, and both the donor and acceptor sides of PSII [25].

Depending on the treatment, the effect of NPs was observed at different stages of electron transport in PSII. Fe_2_O_3_-NPs and CeO_2_-NPs mainly affected the final stage of the reduction of electron carriers on the acceptor side of PSI, which was visible as the G band in the I-P phase [61]. On the other hand, SnO_2_-NPs, TiO_2_-NPs and SiO_2_-NPs showed a slight negative effect in the O-J phase, which describes the state of the PSII donor part and provides information on the size and absorption capacity of LHCII as well as communication between PSII reaction centers [27, 62]. The increase in fluorescence observed by us in this phase was associated with a slight, temporary decrease in the efficiency of energy transport between the antenna complexes and the PSII active center. This is usually associated with changes to the thylakoid membrane structure and reorganization of PSII units [62, 63]. In turn, the rapid increase in fluorescence in the I-P phase seen in all plants (treated and control) may be the result of the elevated temperature at which the photosynthetic activity was measured.

It should be emphasized that the methods used revealed subtle changes at the level of PSII functioning, which were not visible during the analysis of ChlF parameters, but indicated a possible mechanism of the nanoparticles interaction. In this case, despite the presence of the so-called “stress bands”, the induction of plant stress caused by NPs could not be found, based on this measurement method, especially since the observed changes were temporary, as shown in Fig. 4A-D. However, it is worth noting that L- and K-band are not unique to stress responses. They were also observed, for example, during changes in energy distribution in PSII related to the reproduction and generative development in ferns [64]. In the present experiment, they proved helpful in identifying the stages of photosynthesis light phase most dependent on nanoparticles.

NPs have a tendency to modify photosynthetic efficiency, photochemical fluorescence, and quantum yield [23]. NPs may affect the photosynthetic performance mainly by light-dependent (Hill reaction) and light-independent (Calvin cycle) reactions, reflecting from changes in PSI and PSII functioning [65, 66]. The photosynthetic efficiency may arise or fall from the regulation of key photosynthetic enzymes, i.a. Rubisco, Rubisco activase, fructose-1,6-bisphosphate phosphatase (FBPase), ribulose-5-phosphate kinase (RBPase), and NADP-glyceraldehyde-3-phosphate dehydrogenase (GPDHase), and phospho*enol*pyruvate carboxylase (PEPC) [12]. All these proposed mechanisms show multilateral effects on photosynthetic process. Nevertheless, the effects of NPs on photosynthesis differ in various plants at species level. We suggest that NPs apply to the plants penetrate leaf cuticules, they can enter into cell walls and membranes and they move into cell cytoplasm. In the cell they can penetrate the plant chloroplast. A detailed explanation of the mechanism of the NPs applied in our study will require further research, e.g. transcriptomics and interactions between the membranes and NPs.

## Conclusions

Nanoparticles caused morphological changes on the leaves of treated oakleaf lettuce, but over time plants managed to rebuild the vast majority of damaged tissues and return to the original morphological state of the leaves. The data presented in this report showed that many reflectance indices and chlorophyll fluorescence parameters changed due to NPs treatment, however, it should be emphasized that the intensity of the plant-nanoparticles interaction and the direction of this interaction depended on the type of nanoparticles applied to the plants and, in ther case of ChlF, measurement time. The JIP test indicated that the treatment with NPs caused changes within the PSII antenna, manifested by a slowdown in the transport of electrons between the Chl molecules of the light-harvesting complex II (LHCII) and the active center of PSII (L- and K-bands). The findings of our research revealed that nanoparticles have a significant impact on the function of the photosynthetic apparatus, however, more studies on the effects of NPs in different plant species are needed to describe underlying mechanism in details.

## Materials and methods

### Plant material

Oakleaf lettuce (*Lactuca sativa* L. var. *foliosa* Bremer) seedlings cv. Kiribati were obtained from Krasoń – Group of Vegetable Seedling Producers (Piaski, Poland). Seeds were supplied by Rijk Zwaan Polska Sp. z o.o. (Warsaw, Poland). Oakleaf lettuce plants were grown in cubic peat pots of 64 cm^3^ volume each placed together in plastic boxes (150 pots per one plastic box). Seedlings were placed on the table in the greenhouse of the University of Agriculture in Krakow (Poland), irrigation was performed daily by flooding the table with tap water, up to ¾ height of the pots, without wilting the shoots. Air temperatures were maintained at the level of 25/20 °C (day/night), on average, relative humidity was ca. 70%, day length was 16 h, light was natural. No additional fertilization was applied during this time. After the plants had reached the 4-leaf stage, the application of nanoparticles (NPs) was done.

### Characterization of nanoparticles

All nanoparticles were purchased from PlasmaChem GmbH (Berlin, Germany) as aqueous colloidal suspensions. Metal nanoparticles (Ag and Au) were obtained in the form of ca. 0.10 mg cm^−3^ (Ag) and 0.05 mg cm^−3^ (Au) colloidal suspension in water with citrate as stabilizer. Average Ag and Au particle size was ca. 10 nm and ca. 20 nm, respectively. Metal/metalloid oxide nanoparticles: cerium oxide (CeO_2_-NPs), iron(III) oxide (α-Fe_2_O_3_-NPs), silicon dioxide (SiO_2_-NPs), tin(IV) oxide (SnO_2_-NPs), and titanium(IV) dioxide (TiO_2_-NPs) were delivered as 5 wt% aqueous suspension with the exception of SiO_2_-NPs (30 wt%). The average particle size was estimated on CeO_2_-NPs – 4 nm, Fe_2_O_3_-NPs – 6 nm, SiO_2_-NPs – 10 nm, SnO_2_-NPs – 6 nm, and TiO_2_-NPs – 6 nm. Anatase phase of Ti and hematite phase of Fe were used.

### Experimental design

Plants were randomly divided into eight groups, each assigned to a different foliar treatment. The NPs concentrations were 6% TiO_2_, 6% SiO_2_; 3% CeO_2_, 3% SnO_2_, 3% Fe_2_O_3_; 0.004% (40 ppm) Ag; 0.002% (20 ppm) Au. The NPs were prepared by dispersing them in deionized water and applied on the plants after 30 min of ultrasonic bath. At the same time, seedlings sprayed with deionized water were considered as control. In each treatment, there were two plastic seedling boxes with 150 plants per box (300 plants per treatment); particular suspensions were applied evenly on the leaves, only once, in a dose of 50 cm^3^ per box (ca. 0.33 cm^3^ per plant). Hand sprayer equipped with a nozzle giving a fine droplet fall was used. Final concentrations were chosen based on preliminary tests in which the external phytotoxicity symptoms (necroses, leaf deformations) caused by NPs applied in various concentrations were assessed and on the basis of data obtained in other experiments [36, 37]. The very low concentrations of Ag and Au used in the present experiment, compared to metal(oid) oxides, resulted from a very small amounts of metallic nanoparticles of the commercial product offered by PlasmaChem GmbH.

### Evaluation of the optical properties of leaves

The reflection of light radiation was determined in the range of 400–1000 nm using a CID Bio-Science CI-710 spectrometer (Camas, WA, USA) with the SpectraSnap software (CID Bio-Science). Leaf measurements were performed 9 days after the NPs treatment. Based on the analysis of the reflection spectra, the reflection coefficients were calculated for: anthocyanins [ARI2 = (R_550_^−1^ − R_700_^−1^) × R_800_ [67], flavonols [FRI = (R_410_^−1^ − R_460_^−1^) × R_800_ [68], and water [WBI = R_900_ × R_970_^−1^ [69]. Additionally, the ratio of carotenoids to chlorophyll *a* content [SIPI = (R_800_ − R_445_) × (R_800_ + R_680_)^−1^ [70] was calculated and the value of the index associated to photosynthetic efficiency [PRI = (R_531_– R_570_) × (R_531_ + R_570_)^−1^ [47]) was determined. The letter R in the equations denotes the intensity of reflection at the radiation wavelength given in subscript.

### Chlorophyll *a* fluorescence measurements

The photosynthetic activity of photosystem II (PSII) was characterized by the parameters of chlorophyll *a* fast fluorescence measured with a Plant Efficiency Analyzer (PEA, Hansatech Instruments Ltd., Norfolk, UK) according to its manual. Plants were kept in the greenhouse. The fluorescence parameters of the plant leaves were measured on the upper side of the leaf blade, between the main and lateral veins. The clips with a 4 mm diameter hole were clamped on the leaf to be tested for 20 min dark adaptation. Radiation of 3 mmol (quantum) m^–2^ s^–1^ was used for the excitation of chlorophyll fluorescence. Measurements were performed four times: 3, 5, 7 and 9 days (t1, t2, t3, t4, respectively) after the NPs treatments. The following parameters were calculated [47]: the maximum photochemical efficiency of PSII (F_v_/F_m_), the maximum efficiency of the water splitting complex on the PSII donor side (F_v_/F_0_), and a photosynthetic performance index describing the vitality of PSII (PI_ABS_). Energy flow through PSII was evaluated on the basis of the flow parameters: ET_0_/RC – rate of electron transfer through active reaction centre (RC), DI_0_/RC – total energy dissipation, not trapped by RC, and light absorption flux (for PSII antenna chlorophylls) per PSII reaction center (ABS/RC). The parameter 1 – V_I_ is interpreted as the efficiency/probability by which electrons move from PSII to PSI acceptor side.

The fast Chl *a* fluorescence kinetics (O-J-I-P) was measured by PEA fluorimeter, analysed in PEA Plus program (Hansatech Instruments Ltd., Norfolk, UK) and elaborated with Microsoft Excel 2010 (Microsoft, Redmond, WA, USA). The following fluorescence intensity measurement points were adopted for the O-J-I-P test: O – 20 μs, J – 200 μs, I – 20 ms, P – 200 ms. Differential characteristics of changes in the kinetics of fluorescence increase for individual sections of the O-J-I-P curve (O-K, O-J, J-I, I-P) were calculated by subtracting the normalized (to points O and P) fluorescence values of plants treated with nanoparticles from the normalized values obtained for the control.

### Statistical analysis

Statistical analysis was performed with the Statistica 13.3 package (TIBCO Software Inc., Palo Alto, CA, USA). Differences between metal/metal(oid) oxides nanoparticles treatments and control within particular time point were analysed by Duncan's test. Significance between means for ChlF parameters were checked at *p* ≤ 0.05 (*), *p* ≤ 0.01 (**) and *p* ≤ 0.001 (***). Homogeneous groups were determined at the significance level *p* ≤ 0.05. Single-point chlorophyll fluorescence data represent the mean of 20 measurements per each treatment grouped in four replications ± standard deviation (SD). Leaf reflection analysis was performed as the mean for the young leaf and the older leaf of the given plant in a treatment (10 plants in total per treatment).

## Data Availability

The datasets used and analyzed during the current study are available from the corresponding author on reasonable request.

## Abbreviations

1 – V_I_: The efficiency/probability by which electrons move from PSII to PSI acceptor side
ABS/RC: Absorption flux (of antenna Chls) per RC
ARI2: Anthocyanin Reflectance Index 2
DI_0_/RC: Total energy dissipation not trapped by the PSII reaction center
ET_0_/RC: Rate of electron transfer by the active PSII reaction center
FRI: Flavonol Reflectance Index
F_v_/F_m_: Maximum quantum yield of PSII
F_v_/F_0_: Maximum efficiency of the water-splitting reaction of the donor side of PSII
NPs: Nanoparticles
O-J-I-P: Fast chlorophyll fluorescence induction curve
PI_ABS_: Performance index
PRI: Photochemical Reflectance Index
RC: Reaction center
SIPI: Structure-insensitive pigment index
WBI: Water Band Index
